# Endovascular management of contralateral gate maldeployment during EVAR: Case report of interventional technique

**DOI:** 10.1016/j.ijscr.2021.02.023

**Published:** 2021-02-09

**Authors:** Samer Koussayer, Aseel Abuduruk

**Affiliations:** a Vascular Surgery Department, King Faisal Specialist Hospital and Research Center, Makkah Al Mukarramah Branch Rd, Al Mathar Ash-Shamali, 1121, P.O. Box: 3354, Riyadh, Saudi Arabia; b Vascular Surgery, Taif University, College of Medicine Taif University, Alseteen Street, Alhaweyia, Al-Taif, 21944, B.O. Box 11099, Saudi Arabia

**Keywords:** EVAR, Endovascular Abdominal aortic Aneurysm Repair, CLG, Contra-Lateral Gate, CABG, Coronary Artery Bypass Graft, CTA, Computed Tomography Angiogram, Contralateral gate, Endovascular repair, Infrarenal aortic aneurysm, Maldeployment, Case report

## Abstract

**Introduction:**

Endovascular repair of infra renal aortic aneurysm is becoming the preferable method of intervention compared to open surgery due to the lower early morbidity rates. One of the complications during the procedure is contra-lateral gate (CLG) maldeployment. Learning the endovascular salvage options is necessary to avoid the morbidities of open surgical conversion.

**Presentation of case:**

we present a case of maldeployment during an endovascular exclusion of aortic aneurysm in a high cardiac risk patient for whom an endovascular management was successful.

**Discussion:**

We discuss the detailed technical aspect in managing CLG maldeployment in our case as well as variable options from literature.

**Conclusion:**

Despite the available possibility to convert to open, there are multiple endovascular bail out alternatives to manage maldeployment that can be successfully performed by experienced interventionalist.

## Highlights

• Endovascular repair of infra-renal aortic aneurysm is becoming the preferable method of intervention.
• Contralateral gait cannulation is an important, challenging step during infra-renal aortic aneurysm endovascular repair.
• Maldeployment of the contralateral gait outside the aortic sac is a difficlut situation that mandates urgent intervention.
• Conversion to open surgery is the main solution of maldeployment, but several endovascular salvage methods exist.

## 1 Introduction

The most important factor to predict a successful EVAR is proper planning. Identifying anatomical difficulties is mandatory to avoid unpleasant surprises. Neck angulation, iliac tortuosity, and thrombus load can have an impact on the procedure [1]. Adequate knowledge of the chosen device design (bimodular/trimodular) and its possible pitfalls cannot be over emphasized.

Completion of EVAR requires CLG catheterization, a major step that could be achieved easily or could be quite challenging [2]. If the wire is misplaced and the surgeon proceeded with final deployement, not recognizing this technical error, maldeployment will occur in the aortic sac. If not diagnosed intra operatively, maldeployment can result in serious complications [3]. Open conversion is an option but it precludes increased perioperative mortality and morbidity.

Our case presents a unique uncommon complication that is specific for tri-modular devices and discusses the endovascular options of salvage. This intervention was performed in King Faisal Specialist Hospital and Research Center which is a community tertiary center. This work has been reported in line with the SCARE as well as PROCESS criteria [4,5].

## 2 Case presentation

A 74-year-old gentleman who was referred to vascular surgery clinic with a 9-cm infra-renal abdominal aortic aneurysm and mild back pain. He is known to have hypertension, coronary artery disease, with history of CABG. He was declared as a high risk for open repair by cardiologist, so percutaneous elective EVAR under local anesthesia was planned. His family and psychosocial history were unremarkable.

A low-profile tri-modular device was chosen because of his small iliac arteries and tortuosity. The procedure was performed by a senior vascular surgery consultant with immense experience in endovascular interventions.

The EVAR was started with bilateral femoral percutaneous access. The main body of the endograft was deployed successfully (right femoral access) with its both short limbs facing anterioposterior position due to the severe iliac tortuosity. The CLG was cannulated without difficulty and the position inside the main body of the graft was confirmed by twisting the pig tail catheter inside.

**Fig. 1:**
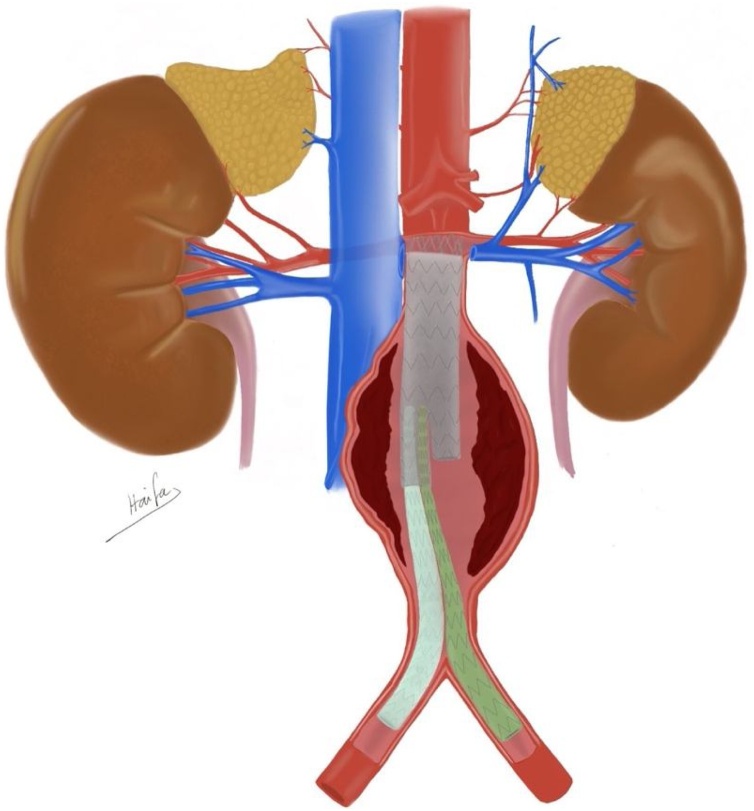
Maldeployment of both iliac limbs in to the ipsilateral gate in a trimodular design.

According to the fluoroscopy images, the location of the wire seemed to be in the correct place so deployment of both iliac limbs was performed. Completion angiogram revealed maldeployment of both limbs in to the ipsilateral gate (Fig. 1).

**Fig. 2:**
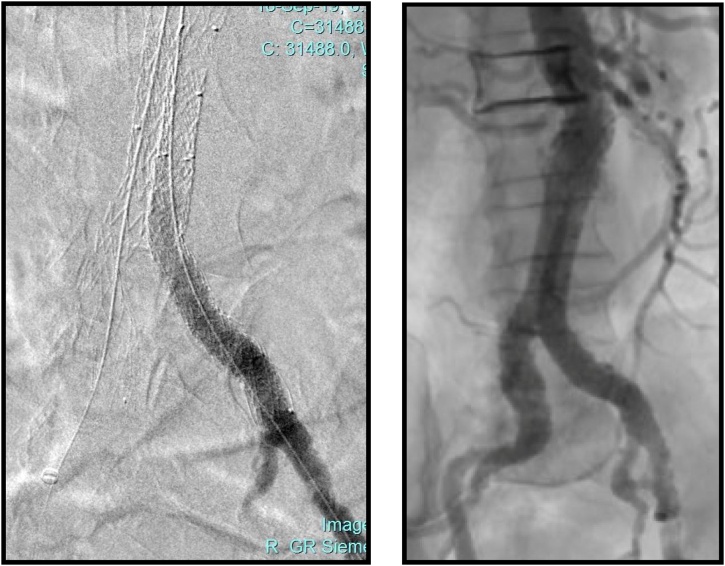
The final angiogram images of our patient after balloon expandable covered stents deployment crushing the left iliac extension stent graft of EVAR.

After recognition of the problem we proceeded endovascular salvage. Cannulation of the contra lateral gate was achieved via a retrograde access from the left femoral artery. Hydrophilic wire was manipulated between the maldeployed left iliac limb and the native common iliac artery with the help of Vertebral catheter until we accessed the aneurysmal sac. Another wire was passed and crossed over the flow divider from the ipsilateral limb (right femoral artery). This wire was snared from the left femoral access. Afterwards, three balloon expandable covered stents were deployed starting from the flow divider of the main stent graft all the way down the left CIA bifurcation. The maldeployed limb was squeezed between both iliac stent grafts using kissing balloon technique (Fig. 2).

**Fig. 3:**
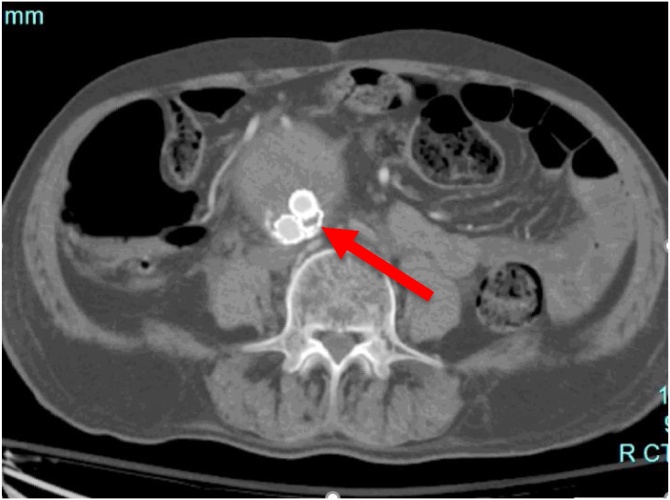
CTA follow up prior to hospital discharge showing the crushed maldeployed stent (RED arrow) and the other patent covered stent. No evidence of endoleak.

**Fig. 4:**
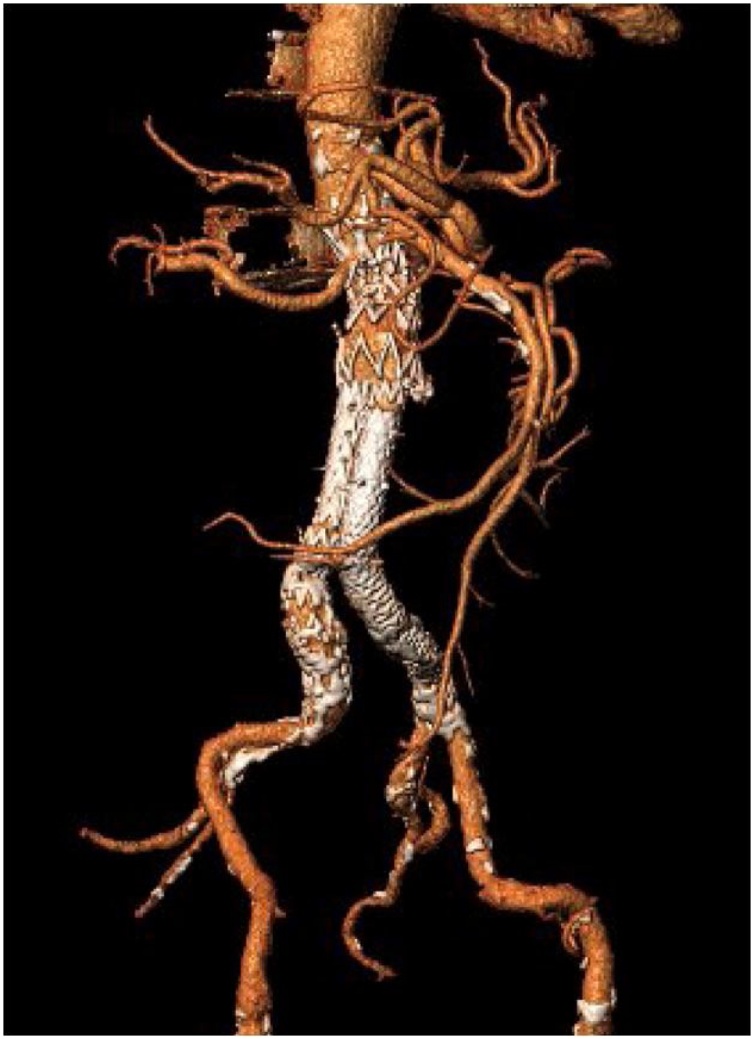
Post EVAR3D reconstruction of the previous follow up CTA.

Patient recovered from the procedure without complications and discharged home after two days. He was placed on dual antiplatelets therapy for 6 months. At 3-, 6-, and 12-months follow-up, the patient was symptom free and had good distal pulses on physical examination. Computed tomography angiogram (CTA) follow-up revealed complete exclusion of the aneurysm sac, no endoleak, decreased aneurysm size, and patent bilateral EVAR limbs (Fig. 3, Fig. 4). The patient was instructed to continue aspirin for life and educated about the importance of an annual CTA follow-up.

## 3 Discussion

Endovascular abdominal aortic aneurysm repair is a common procedure in vascular surgery [6]. Recognition of intraoperative challenges is important to prevent procedural complications. A stent graft device is deployed during EVAR to exclude the aneurysmal sac. Variable designs are available with multiple components (bi-modular and tri-modular designs) and selection depends on preoperative planning [7].

Cannulation of the CLG is a major step for a successful EVAR. It can be accomplished within few minutes, or sometimes may be challenging [2,8]. Multiple anatomical factors contribute to difficulties in CLG cannulation like a large aneurysm with little thrombus, narrowed residual aortic lumen with large thrombus load, or severe aortic neck / iliac arteries tortuosity. Device’s design can influence the ease of CLG cannulation. Wire misplacement anywhere outside the CLG of the main body stent graft can result in subsequent improper deployment of the endograft (maldeployment). Aneurysmal sac is not successfully excluded in this complication and the risk of rupture is high if not recognized and managed intraoperatively [9].

Confirmation of correct wire location is necessary prior to deployment of the contralateral limb and can be performed using variable methods (Moulding technique or the Pig tail rotation) [10,11]. Ren-Fu Shie et al. has described a step-by-step method to facilitate cannulation of the CLG in 83 patients during which the orientation of the image intensifier was manipulated. Average time to cannulation was 22.88 s (range 1–174 s) [12]. Turret technique describes the use of a reverse curve catheter and manipulate it via the end of a long sheath to allow a 360° steering toward the gate [13]. An antegrade access can achieve successful CLG catheterization in a shorter time avoiding prolonged radiation exposure and operative time. Titus et al. had prospectively studied 101 EVARs comparing cannulation times in retrograde versus antegrade cannulation with snare. Median cannulation times were similar (2.7 and 3.9 min respectively), however, the author mentioned that if retrograde cannulation was not achieved in 5 min, the chances of eventual success decreased significantly, and crossover with snare was more efficient [14]. Brachial access remains another bail out technique but with frequent local access complications [15].

Maldeployment is a rare technical complication that is preventable by following the mentioned techniques. When it occurs, the contralateral limb endograft is usually deployed in the aneurysmal sac. Two cases of maldeployment of the CLG within the sac were described in literature and salvaged endovascularly [11,16]. Our case is unique in that maldeployment occurred within the ipsilateral limb of the main body endograft which will result in failure of the procedure as well as subsequent lower limb ischemia. We used a tri-modular endograft which has a shorter limb compared to the bimodular design and this might have contributed to the development of this situation. The wire has missed the CLG and passed in to the ipsilateral limb. Pig tail catheter twisting method had confirmed the location of the wire being inside the main body of the endograft (but not inside the CLG) which resulted in deployment of both iliac limbs within the ipsilateral gate.

Although conversion to open surgery is an option but it carries a considerable risk in a cardiac patient. Variable endovascular techniques can be performed by an experienced interventionalist to salvage this complication including:

1 Use of aorto-uni-iliac stent graft configuration with femora-femoral bypass.
2 Plugging the CLG from brachial access and performing femoral-femoral bypass.
3 Cannulation of the CLG through the ipsilateral femoral access and cross over, passing a wire between the maldeployed graft and native artery then snare it distally and deploy a new contralateral limb.
4 Gain access to CLG via an antegrade access (contralateral femoral artery), pass the wire between the maldeployed graft and the native artery, then snare it from the ipsilateral limb over the flow divider, and deploy a new contralateral limb. This was performed in our case. We found balloon expandable stents superior in radial force and durability within a crowded iliac system compared to the conventional softer iliac extension limbs. No cases of maldeployment of both iliac limbs in the ipsilateral gate were found in literature.

## 4 Conclusion

Maldeployment of both iliac limbs inside the ipsilateral gate is a unique complication associated mainly with tri-modular devices. Once identified, open conversion is the best option in good risk patients. There is multiple Endovascular bail out alternatives in high-risk patient and with experienced endovascular interventionalist. The long-term outcomes of these techniques need to be validated.

## Declaration of Competing Interest

The authors report no declarations of interest.

## Funding

No funding was required.

## Ethical approval

No ethical approval was needed.

## Consent

Written informed consent was obtained from the patient for publication of this case report and accompanying images. A copy of the written consent is available for review by the Editor-in-Chief of this journal on request

## Author contribution

1 Samer Koussayer: Conception and Design, data collection, critical revision, manuscript approval, Agreement to be Accountable.
2 Aseel Abuduruk: Data collection, Writing the Manuscript, manuscript approval, Agreement to be Accountable.

## Registration of research studies

Not Applicable.

## Guarantor

Samer Koussayer

## Provenance and peer review

Not commissioned, externally peer-reviewed.
